# Evaluation of Different EEG Acquisition Systems Concerning Their Suitability for Building a Brain–Computer Interface: Case Studies

**DOI:** 10.3389/fnins.2016.00441

**Published:** 2016-09-30

**Authors:** Andreas Pinegger, Selina C. Wriessnegger, Josef Faller, Gernot R. Müller-Putz

**Affiliations:** ^1^Institute of Neural Engineering, Graz University of TechnologyGraz, Austria; ^2^Laboratory for Intelligent Imaging and Neural Computing, Columbia University in the City of New YorkNew York, NY, USA

**Keywords:** brain–computer interface, practical electrodes, dry electrodes, water-based electrodes, gel electrodes, P300, electrode test

## Abstract

One important aspect in non-invasive brain–computer interface (BCI) research is to acquire the electroencephalogram (EEG) in a proper way. From an end-user perspective, it means with maximum comfort and without any extra inconveniences (e.g., washing the hair), whereas from a technical perspective, the signal quality has to be optimal to make the BCI work effectively and efficiently. In this work, we evaluated three different commercially available EEG acquisition systems that differ in the type of electrodes (gel-, water-, and dry-based), the amplifier technique, and the data transmission method. Every system was tested regarding three different aspects, namely, technical, BCI effectiveness and efficiency (P300 communication and control), and user satisfaction (comfort). We found that water-based system had the lowest short circuit noise level, the hydrogel-based system had the highest P300 spelling accuracies, and the dry electrode-based system caused the least inconveniences. Therefore, building a reliable BCI is possible with all the evaluated systems, and it is on the user to decide which system meets the given requirements best.

## 1. Introduction

Measuring electrical activity of the human brain and utilizing this data to bypass the traditional motor output pathways of the nervous system is one of the main purposes of brain–computer interface (BCI) systems. One way to collect these signals non-invasively is by using electroencephalography (EEG). Nowadays, two main factors that impede the widespread use of BCIs for healthy as well as for severely impaired people are the BCI control method (i.e., how measurable brain signals are generated) and the EEG signal acquisition system (i.e., the used hardware) to measure the signals.

We consider three control methods based on different brain signals: (i) neural oscillations, (ii) event-related potentials (ERP), and (iii) steady-state evoked potentials (SSEP).

A typical BCI based on neural oscillations, for example, utilizes the fact that defined frequency components of the EEG signal create a typical pattern briefly before, during, and after movement execution and less pronounced at movement imagination (e.g., Pfurtscheller et al., 2000; Faller et al., 2014; Schwarz et al., 2015). Tasks that also show detectable oscillations are word association, mental subtraction, mental rotation, auditory imagery, or spatial navigation (Friedrich et al., 2013). This phenomenon can be used to create a so-called self-paced BCI (i.e., no external trigger is needed). However, the illiteracy rate and also the effort on training the system are very high (cf. Blankertz et al., 2010).

The other two BCI control methods need stimulation to evoke a defined pattern in the EEG. A very prominent representative of this group relies on the P300 EEG-wave complex. This positive amplitude approx. 250–500 ms after an event can be elicited by an odd-ball paradigm (Pritchard, 1981; Polich, 2007). Due to the fact that the difference between the P300 amplitude and the spontaneous EEG is small, the stimulation has to be repeated, and the signals averaged until the signal to noise ratio (SNR) is high enough for classification. One of the first implemented BCIs (Farwell and Donchin, 1988) was based on this method. Many studies were conducted to show that P300-based BCIs enable healthy as well as motor impaired users to communicate or to control their environment (Donchin et al., 2000; Piccione et al., 2006; Hoffmann et al., 2008; Nijboer et al., 2008; Kaufmann et al., 2013; Pokorny et al., 2013).

SSEP-based BCIs, as another type, also require stimulation. The stimuli are periodically presented at a repetition rate higher than approx. 6 Hz. SSEP BCIs are based on the fact that the stimulation rate is represented as SSEP (i.e., a periodically repeated pattern) in the EEG when the user shifts their attention to these stimuli. Stimuli can be visual (SSVEP, Bagolini et al., 1988; Müller-Putz et al., 2005; Vialatte et al., 2009), auditory (Stapells et al., 1984; Picton et al., 2003; Lopez et al., 2009), or somatosensory (Müller-Putz et al., 2001, 2006; Pokorny et al., 2011).

The second important part of each BCI is how brain signals are measured. At the very beginning, in 1924, scientists inserted steal needles into the subcutaneous tissue of the scalp and had galvanometers to visualize and interpret the recorded signals (Berger, 1929). The quality and the interpretability of the signals improved with the usage of vacuum tubes, and later, transistor technology was used to amplify the very small signals. Silver chloride (AgCl) covered electrodes, which are standard nowadays, were introduced by Berger in 1931 (Collura, 1993). Today, Bergers' method would not be called non-invasive but it is called minimal invasive EEG acquisition, because he penetrated the skin of the scalp. More invasive brain signal acquisition techniques are the electrocorticogram (ECoG), subdurally/epidurally measured on the brain surface (Leuthardt et al., 2004), and multi/single unit activity derived with needle electrodes directly from the cortex (Hochberg et al., 2006). However, these methods are more common in clinical settings and not yet envisaged for everyday use in practical BCI systems.

One major issue concerning the EEG measurement is noise. According to Bressler and Ding (2006), the following sources of noise in brain activity recordings exist: (1) potentials from the brain (cephalic noise), (2) potentials from the head muscles and skin, eyes, and tongue (extracephalic cranial noise), (3) potentials from parts of the body other than the head, such as the heart (extracranial physiological noise), (4) random microscopic fluctuations at the electrodes (thermal noise), (5) noise from movement of the person or animal (movement artifact), (6) fluctuations introduced by electronic recording components (electronic noise), (7) radiated contamination from other electrical equipment (environmental noise), and even (8) fluctuations due to imprecision in the discrete digitization of the continuously varying voltage from the electrode for storage in a digital computer (quantization noise) (Bressler and Ding, 2006). According to points 4–8, the recorded amount of noise strongly depends on the characteristics of the EEG acquisition system being used.

To measure EEG, a way has to be found to bridge the gap between the electrode and skin surface. Currently, there are three common types of electrodes that differ based on whether the conductive connection is established based on gel, water, or no additional conductive substance (“dry”).

The gel-based type can be subdivided based on the usage of abrasive gel and hydrogel, respectively. Abrasive gel is mainly used in combination with passive electrodes (i.e., no amplification happens directly at the electrode). In contrast, the hydrogel is mainly used for active electrodes (i.e., the signal is pre-amplified directly at the electrode). The main difference between these two types of gels is that with the abrasive gel, the topmost layer of the skin, consisting of dead cells, is removed in a time-consuming procedure to decrease the impedance. This can lead to skin irritation, infection, or inflammation. For both types of gel-based electrodes, gel has to be filled between the electrode and the scalp, which then typically makes it necessary for the user to wash their hair, after the measurement. Water-based electrodes use a water or saline solution soaked felt or fabric to connect the metal part of the electrode with the skin. Using tap water-soaked fabric to connect the two surfaces is a new and practical method. This type of electrodes should deliver a very good signal quality, and no hair wash is needed after the measurement (Volosyak et al., 2010).

Dry electrodes, work without any conductive substance. Pins made of metal alloy or conductive rubber are pressed directly onto the skin, and rely on small amounts of existing perspiration to get connected to the skin. Several studies were conducted highlighting the advantages of different dry electrode-based systems (e.g., Zander et al., 2011; Guger et al., 2012; Mota et al., 2013). However, experience shows that one main disadvantage of this type of electrodes is their sensitivity to movement artifacts.

Several papers deal with user-centered BCI approaches (e.g., Zickler et al., 2011; Kübler et al., 2014; Scherer et al., 2015). Concerning data acquisition, these papers find similar results: Users want to have an easy to handle data acquisition system, which should not require the subject to wash their hair after acquisition. At the same time, the signal quality should allow for BCI accuracies comparable to gel-based systems. The dry electrode-based systems can only fulfill the first part as the BCI accuracies of gel-based systems are not fully achieved yet. For example, Zander et al. (2011) reported a mean BCI classification accuracy of 94.0% for gel-based and 90.7% for dry electrode-based systems, respectively, and Guger et al. (2012) reported a mean P300 BCI accuracy of 91.0% for gel-based and 90.4% for dry based-electrode systems, respectively.

Existing literature only compared one new EEG acquisition system (i.e., the dry electrode-based or the tap water-based electrode system) with one gel-based system, aiming to show that the new system works comparably well (cf. Volosyak et al., 2010; Zander et al., 2011; Guger et al., 2012; Mota et al., 2013).

The aim of this study is to evaluate three different EEG acquisition systems with regard to their suitability for building a BCI, meeting technical requirements and requirements for user-centered design specifications. Therefore, we tested and evaluated a hydrogel-based, a tap water-based, and a dry electrode-based systems with their corresponding amplifiers under controlled conditions. Technical tests and real BCI tasks with healthy volunteers were performed. Subsequently, we compared our findings with the findings of existing literature.

## 2. Materials and methods

### 2.1. Systems and data acquisition methods

Three different EEG acquisition systems were tested. The systems differ in electrode design, amplifier technique, and data transmission (see Table 1).

**Table 1:** **Comparison of the used EEG amplifier systems**.

	**g.GAMMAsys**	**Mobita**	**g.Sahara**
Electrode technique	Hydrogel-based	Tap water-based	Dry
ADC resolution	24 bit	24 bit	24 bit
Voltage input range	±250.0*mV*	±204.8*mV*	±250.0*mV*
Notch filter	50 and 60 Hz	n/a (active cable shielding)	50 and 60 Hz
Sampling frequencies	64−38 400 Hz	250–2000 Hz	64–38 400 Hz
Data transmission technique	USB	WiFi (802.11b/g)	USB

#### 2.1.1. The hydrogel-based electrode system

**Figure 1:**
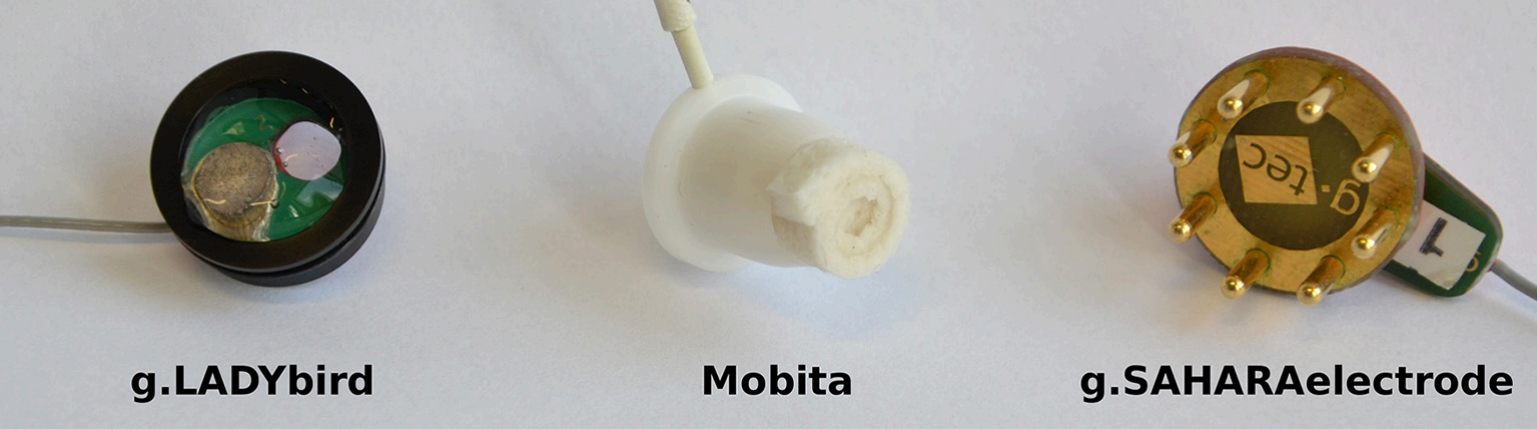
**From left to right: the g.LADYbird hydrogel-based electrode, the tap water-based electrode of the Mobita system, and the dry electrode of the g.Sahara system**.

We tested the g.GAMMAsys from g.tec (Guger Technologies OG, Graz, Austria) in combination with active, hydrogel-based, silver/silver chloride (Ag/AgCl) electrodes (g.LadyBirds) (see Figure 1). The system allows the acquisition of up to 64 biosignals such as EEG, electrooculogram (EOG), electromyogram (EMG), and electrocardiogram (ECG) simultaneously in combination with up to four g.USBamps to amplify and transmit the signals via universal serial bus (USB) to a personal computer (PC) or laptop. Main technical specifications are listed in Table 1. In addition, different filter settings are available. After every usage, the electrodes as well as the cap have to be washed under running water using a brush.

#### 2.1.2. The water-based electrode system

The Mobita is a wireless system of the company TMSi (Twente Medical Systems International B.V., Oldenzaal, the Netherlands). It acquires a maximum of 32 channels of EEG plus three channels for the built-in accelerometer. We tested its capability to measure EEG with passive, water-based electrodes (see Figure 1). The special characteristics of these water-based electrodes are rolled-in, tap water-soaked cotton pieces attached to small AgCl pellets as electrodes. These cotton pieces are disposable. Therefore, for regular cleaning, it is sufficient to dry the cap and the wristband. Another main feature is the actively shielded cable connection between the electrodes and the amplifier. This active shielding should strongly reduce the mains interference and cable movement artifacts. These techniques should provide high signal quality without the necessity of washing the hair after the measurement.

Technical specifications are listed in Table 1. The channel bandwidth is limited between direct current (DC) and 0.2 × sampling frequency (i.e., the average number of samples obtained in 1 s). The system uses the WLAN IEEE standard 802.11 b/g to transmit the amplified signals wirelessly to a PC or laptop.

#### 2.1.3. The dry electrode-based system

The g.Sahara is also produced by g.tec (Guger Technologies OG, Graz, Austria). The acquisition of the EEG with up to 16 dry electrodes in combination with the g.USBamp (also from g.tec) is possible. The electrodes consist of 8 pins made of a special gold alloy (see Figure 1). Two different pin lengths (7 and 16 mm) and three different cap sizes are available to adapt the system to different hair lengths and shapes of users' heads. The operator has to find the optimal type of electrodes and cap size for each participant to get the best signal quality. Disposable Ag/AgCl mastoid electrodes are used for reference and ground. The other electrodes have to be cleaned with a smooth cotton cloth and alcohol (70%). Since we used the g.USBamp to amplify the signals, the same technical amplifier specifications for the g.GAMMAsys are valid.

### 2.2. Technical test

**Figure 2:**
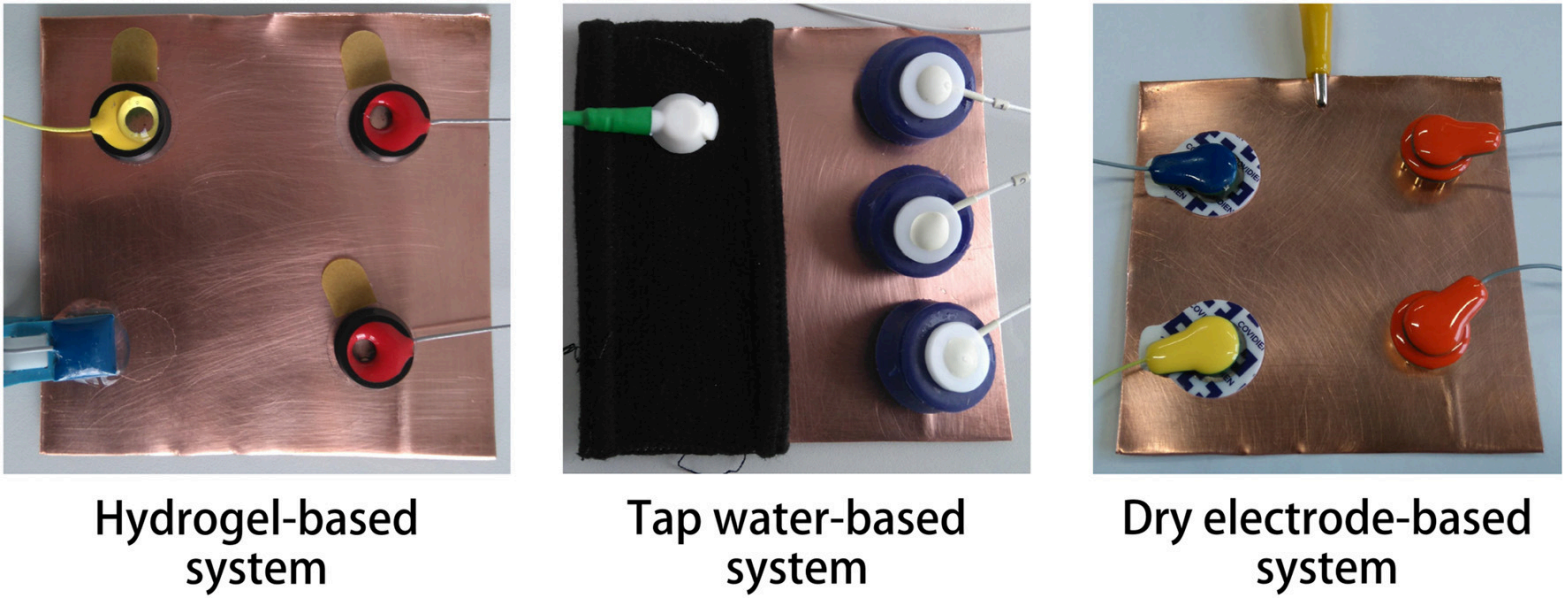
**Setup of the noise test**. All three systems were attached to a polished copper plate to simulate a short circuit of the electrodes.

The short circuit noise of the EEG acquisition systems (electrodes and amplifier) was determined by acquiring the signal of the electrodes that were attached to a polished copper plate (10 × 10 cm) (see Figure 2). The copper plate was polished with an abrasive cleaner and residues were removed with ethyl alcohol shortly before each measurement. The measurement was conducted at normal room temperature (approx. 21°C). Since the electrode systems were purchased nearly at the same time, the influence of aging should be the same for all electrodes.

Sampling frequencies (fs) were 500 Hz for the tap water-based system and 512 Hz for the hydrogel-based and dry electrode-based systems. Signal processing was performed with Matlab (2014b, The MathWorks, Natick, USA). The data of the different electrode systems was streamed to Matlab with the TOBI SignalServer software (Breitwieser et al., 2012). Signal filters were disabled as far as possible to get the full spectrum of the signal. As recommended by the manufacturer, proper grounding was performed for the dry electrode-based system.

Twelve minutes of short circuit noise was recorded with all systems. The first and the last minute were excluded from analysis to avoid any movement artifacts from the operator. Consequently, 10 min of noise were available from all systems for analysis.

The noise was evaluated for a frequency range of 0.1–40 Hz, which is typical for BCI systems. Therefore, the signals were 8th order band-pass filtered (Butterworth) between 0.1 and 40 Hz. The histogram and the amplitude spectrum were calculated with Matlab to compare the different systems. In addition, the root mean square (RMS) was calculated and smoothed with a Gaussian filter (3-dB bandwidth-symbol time = 0.1 s, periods to the filters peak = 4, oversampling factor = 250/256).

### 2.3. User-centered test

BCI effectiveness and efficiency were evaluated with P300 communication and control tasks. Participants had to spell several words and then had to control a multimedia player and a web browser with a P300 BCI. Afterwards, the participants were asked to complete several questionnaires.

It was not possible to randomize the sequence of the tests, because the EEG acquisition systems were not available at the same time. Therefore, the participants tested the dry system first, then the gel-based system, and finally the tap water-based system. However, between the user-centered tests of the different EEG acquisition systems were always more than 1 month, and therefore, one can assume that adaptation or learning effects did not occur.

#### 2.3.1. Participants

Eight healthy volunteers (1 female, mean age 25 ± 2.3, range 22–30 years) participated in this study. All participants stated that they had no history of neurological or psychiatric disorders. The study protocol was approved by the ethics committee of the Medical University of Graz, and the participants gave informed written consent before the experiment. Out of the eight study participants, seven performed the user-centered test per EEG aquisition system.

#### 2.3.2. Signal acquisition and processing

Six channels per system were recorded at a sampling rate of 250 Hz (tap water-based system) and 256 Hz (hydrogel-based and dry system), respectively. The locations of the electrodes (Fz, Cz, Pz, PO7, PO8, and Oz) were based on the extended international 10–20 system for electrode placement. The channels were referenced to the left and grounded to the right earlobe when using the gel-based and dry electrode-based systems. The ground of the tap water-based system was attached to the participant's wrist. In addition, as recommended by the manufacturer, a grounding of the user and operator was performed for the dry electrode-based system. The data acquisition was started only once before each session.

Due to the fact that different data acquisition systems (see Section 2.1) were used, the signal processing differed slightly between the systems (see Table 1). We used the integrated 0.1–60 Hz band-pass filter for the hydrogel-based electrode signal and the 0.5–30 Hz filter for the dry electrode-based signal. The dry electrode is more sensitive to person and cable movement artifacts. Therefore, the signals from that electrodes were band-pass filtered between 0.5 and 30 Hz. Further signal processing was performed with Matlab. The data of the systems were streamed to Matlab, see Section 2.2. No band-pass filter is integrated into the tap water-based system. Consequently, we implemented a filter in Matlab. We used a fourth order Butterworth band-pass filter with cut-off frequencies of 0.1 and 60 Hz. The rest of the signal processing and classification was identical between the tested systems.

#### 2.3.3. P300 classification

A stepwise linear discriminant analysis (SWLDA) classifier was trained with the training data and used for the online runs. The number of flashing sequences (one sequence means that all characters of the stimulation matrix flashed once) was automatically set between 8 and 15 according to Pinegger et al. (2013). The algorithm classifies the training letters with a leave one letter out cross-validation and calculates the reached total accuracy for every flashing sequence. The number of sequences, where 100% accuracy is reached, plus two sequences is chosen as number of flashing sequences for the online run. Whenever 100% accuracy is not reached, but the highest value for the accuracy is higher than 75%, 15 sequences are chosen. Otherwise, the calibration fails and must be performed again.

#### 2.3.4. Experimental design

The participants were seated in a comfortable chair approx. 65 cm away from two computer screens (39.5 and 43 cm diameter). One screen was centered in front of the participants. On this screen, a P300 matrix was displayed to select letters or commands; a second screen was placed right beside the first and was used to show a multimedia player or a web browser. The multimedia player was controlled via network commands (see Halder et al., 2015 for details). The custom-made web browser automatically detects all possible links, buttons, and text fields of the currently shown website and marks them with letters. These letters were sent to the BCI for selection with the P300 matrix. By sending back the desired element to the web browser, the corresponding link, button, or text field was selected (see Halder et al., 2015 for details).

The P300 user interface and the signal processing in Matlab were presented in Pinegger et al. (2013). Elements of the matrix were highlighted with famous faces (Kaufmann et al., 2011).

**Figure 3:**
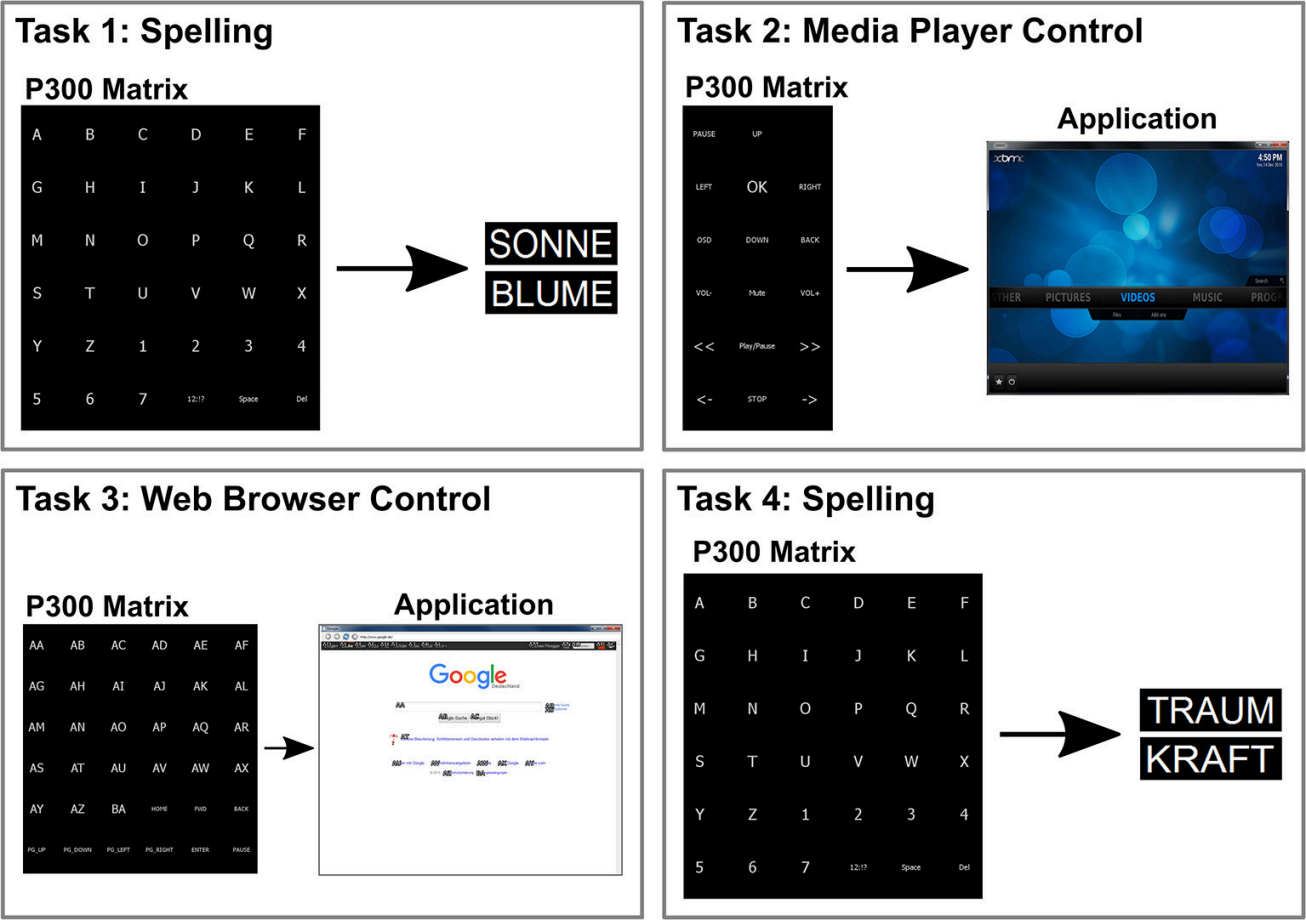
**The participants had to perform four tasks for the user-centered test**. The basis was a P300 BCI system. The first task was to spell two words (ten letters), the second task was to control a media player, the third task was to look for a certain web page in a special web browser, and the fourth task was to spell two words (10 letters) again.

Every participant performed one session per day and system. The experimenter was trained once on every system by an experienced supervisor. In addition, the supervisor supported the experimenter and was available during the whole length of every measurement. A graphical sketch of the user-centered test can be seen in Figure 3. One session comprised the following tasks:

- **P300 classifier training**
  The word “BRAIN” was used for P300 classifier calibration. The speller matrix consisted of six rows and six columns, and every target letter was highlighted 30 times. The collected data was used to train an SWLDA classifier and to calculate an optimal number of flashing sequences.
- **Task 1: First copy spelling**
  The participants had to spell the German words “SONNE” (English “sun”) and “BLUME” (English “flower”) consecutively. Each word was presented to them shortly before they started spelling. The users were instructed not to correct wrongly spelled letters. After a short break, the second word was spelled. The matrix for training and copy spelling was the same.
- **Task 2: Multimedia player**
  Within this task, the participants had to start a slideshow and to look at certain pictures within the Xbox media center (XBMC), a powerful multimedia player. Instructions as to which commands to execute were provided by the experimenter in spoken form. The task could be completed at best with 10 correct selections. To correct wrong selections, the investigator indicated a correct alternative or the way back to the last correct selection. If the goal could not be reached within 15 selections, the task was aborted. The matrix for this task consisted of six rows and three columns.
- **Task 3: Web browser**
  The goal of this task was to navigate to the Wikipedia article about BCI and look over the whole article. The start page was “www.google.de”. Instructions as to which commands to execute were provided by the experimenter in spoken form. The task could be finished within 10–12 correct selections. The ideal number of selections varies because Google has very dynamic web pages, and therefore, the number of links vary considerably over time on these pages. Wrong selections were corrected in the same manner as during the media player run. If the goal could not be reached within 18 selections, the task was aborted. The matrix for this task consisted of six rows and a variable number of columns depending on the number of links on the actual web page. However, the maximum number of columns was seven.
- **Task 4: Second copy spelling**
  This task was performed in the same way as the first copy spelling task. The only difference was that two other words—“TRAUM” (English “dream”) and “KRAFT” (English “force”)—have to be spelled.

Overall, every participant had to perform a minimum of 40 and a maximum of 53 selections per system.

#### 2.3.5. Questionnaires

After the last run of every session, the participants were asked to fill out several questionnaires concerning the satisfaction with the system and the system design.

- VAS: The level of satisfaction of the users was assessed with a visual analog scale (VAS), ranging from 0 (not at all satisfied) to 10 (absolutely satisfied).
- eQUEST 2.0: A usability test was adapted for BCI usage by Zickler et al. (2011). This test evaluates 12 categories (dimension, weight, adjustability, safety, ease of usage, well-being, effectiveness, service features, reliability/robustness, speed, learnability, and aesthetic of design) on a scale from one to five where one stands for not satisfied and five stands for very satisfied. In addition, the three most important categories must be indicated.

#### 2.3.6. Evaluation metrics

The effectiveness was determined by calculating the percentage of correct selections of all selections (accuracy). The efficiency of a system was determined with the amount of flashing repetitions participants needed to make selections with the P300 speller. Results of the questionnaires were evaluated by calculating the averaged values.

## 3. Results

### 3.1. Technical results

**Figure 4:**
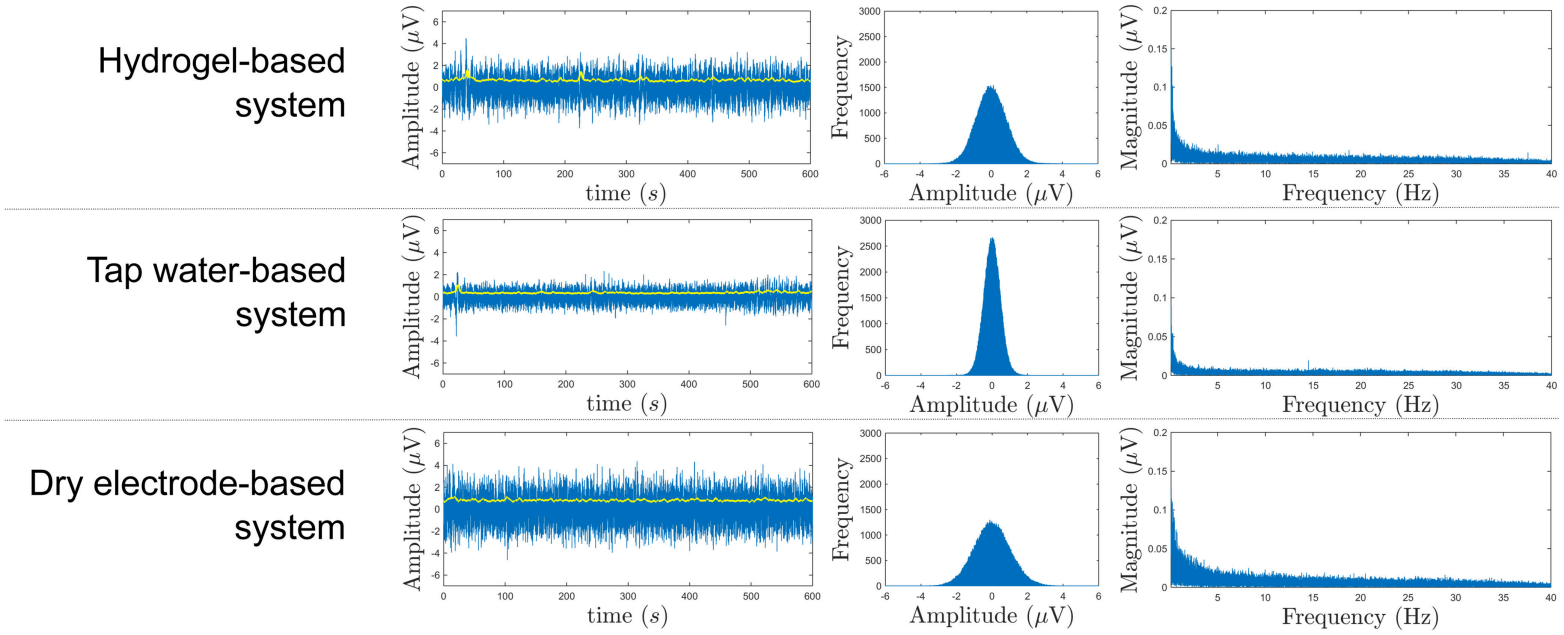
**Signal plot (left), histogram (middle), and amplitude spectrum (right) of the short circuit noise measurement after 0.1–40 Hz band-pass filtering**. The yellow line in the left plots represents the RMS of the signal.

The noise of the different systems was recorded and evaluated. A graphical comparison of the signals from the systems can be seen in Figure 4. The tap water-based system with a mean RMS (yellow line in Figure 4) of 0.37 μ*V* had the lowest measured value followed by the hydrogel-based (0.68 μ*V*) and the dry electrode-based system (0.82 μ*V*) within the frequency range of 0.1–40 Hz. Moderate pressure on the electrodes was necessary to obtain a good signal from the tap water-based and the dry electrode-based system.

### 3.2. User-centered results

#### 3.2.1. Effectiveness and efficiency

**Figure 5:**
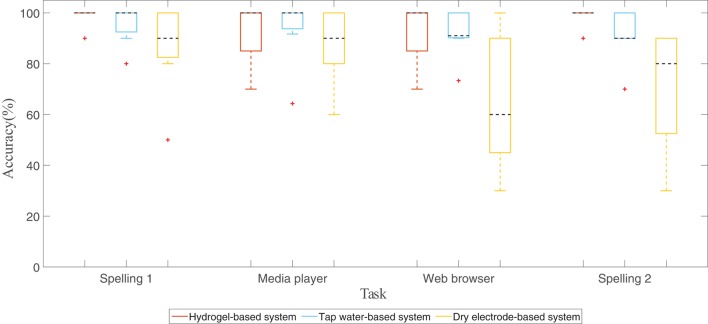
**Boxplots showing the BCI accuracies of each task for every system**. The central mark (dashed line) of each box is the median, the edges of the box are the 25th and 75th percentiles; the whiskers extend to the most extreme data points (1.5 × interquartile range). Outliers are marked with red crosses.

The hydrogel-based system was the most effective with a mean BCI accuracy of 96 % (SD: 3.5) followed by the tap water-based system with 93 % (SD: 4.5) and the dry electrode-based system with 77 % (SD: 11.8). On average, the accuracies of the hydrogel-based (between 93 and 99 %) and the tap water-based system (between 91 and 96 %) stayed stable above 90 % over the four tasks, whereas the dry electrode-based system showed decreasing accuracies over time from 87 % for the first spelling task to 70 % for the second spelling task (see Table 2 and Figure 5).

**Table 2:** **Accuracies of the systems in percent (%)**.

**Participant**	**Hydrogel-based system**				**Tap water-based system**				**Dry electrode system**			
	**Sp1**	**MmP**	**WeB**	**Sp2**	**Sp1**	**MmP**	**WeB**	**Sp2**	**Sp1**	**MmP**	**WeB**	**Sp2**
P1	100	100	70	100	100	100	91	90	80	90	60	50
P2	100	100	100	100	90	100	90	100	90	80	60	90
P3	90	80	100	90	100	100	100	100	50	80	40	60
P4	100	70	80	100	80	64	73	70	90	60	30	30
P5	100	100	100	100	^*^	^*^	^*^	^*^	100	100	100	80
P6	100	100	100	100	100	100	91	100	100	100	100	90
P7	100	100	100	100	100	100	100	90	100	100	60	90
P8	^*^	^*^	^*^	^*^	100	92	100	90	^*^	^*^	^*^	^*^
Mean	99	93	93	99	96	94	92	91	87	87	64	70
SD	4	13	1	4	8	13	10	11	18	15	27	24

Sp1, Sp2… Spelling run 1, 2; MmP…Multimedia player; WeB…Web browser.

* Data not available for this system.

The inter-participant variance (cf. SD in Table 2) was low for the hydrogel-based system, moderate for the tap water-based system, and high for the dry electrode-based system.

**Figure 6:**
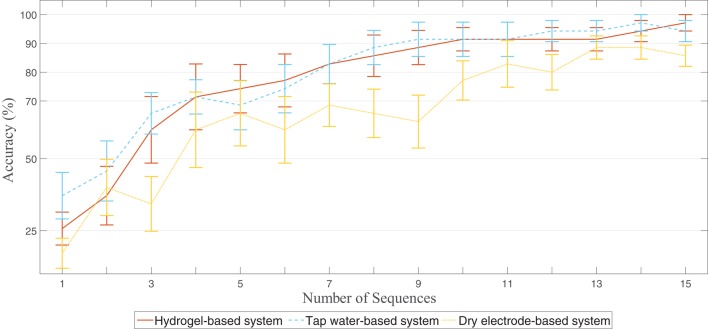
**Result of the leave one letter out cross-validation of the training data**. Error bars indicate the standard error of the mean. The dry electrode-based system showed in general lower accuracies at the same number of sequences compared to the hydrogel-based and the tap water-based systems.

The tap water-based and gel-based systems showed on average the same number of needed sequences followed by the dry electrode-based system (see Table 3). The overall result of the training cross-validation can be seen in Figure 6. The hydrogel-based and tap water-based systems showed comparable results; the accuracies of the dry electrode-based system, however, were slightly lower at the same number of sequences.

**Table 3:** **Sequences needed after training**.

**Participant**	**Hydrogel-based system**	**Tap water-based system**	**Dry electrode system**
P1	8	8	8
P2	15	13	13
P3	14	9	15
P4	13	11	15
P5	8	^*^	8
P6	8	8	15
P7	8	14	15
P8	^*^	10	^*^
Mean	10.6	10.4	12.7
SD	3.3	2.4	3.3

Minimum possible value is eight.

* Data not available for this system.

#### 3.2.2. Satisfaction

Overall device satisfaction per system and results of the eQUEST 2.0 are listed in Table 4. Scores from the VAS were between 6.64 (dry electrode) and 8.76 (tap water-based) on average and indicate a high general satisfaction.

**Table 4:** **Results of the eQUEST 2.0 and VAS for all systems**.

	**Category**	**Hydrogel-based system**	**Tap water-based system**	**Dry electrode system**
	Dimensions	4.6 (0.8)	4.6 (0.8)	4.1 (0.7)
	Weight	4.4 (1.0)	4.6 (0.5)	4.4 (0.8)
	Adjustment: EEG cap	4.4 (0.8)	4.7 (0.8)	4.7 (0.8)
	Adjustment: Amplifier	4.8 (0.4)	4.8 (0.4)	4.8 (0.4)
	Safety	5.0 (0.0)	5.0 (0.0)	5.0 (0.0)
	Comfort: physical	4.1 (0.9)	4.0 (0.8)	4.1 (0.9)
	Comfort: emotional	4.3 (1.0)	4.4 (0.8)	4.3 (1.0)
eQUEST 2.0	Easy of use	4.4 (0.5)	4.6 (0.8)	**3.7 (1.3)**
	Effectiveness	**4.4 (0.5)**	**4.4 (0.8)**	**3.3 (1.5)**
	Reliability: EEG cap	**4.7 (0.8)**	**5.0 (0.0)**	**5.0 (0.0)**
	Reliability: Amplifier	**5.0 (0.0)**	**5.0 (0.0)**	**4.7 (0.8)**
	Speed	**3.4 (1.3)**	**3.7 (1.0)**	**3.6 (1.0)**
	Learnability	4.7 (0.5)	4.9 (0.4)	**4.9 (0.4)**
	Aesthetic design: EEG cap	3.9 (0.9)	4.1 (1.1)	4.0 (1.3)
	Aesthetic design: Amplifier	4.4 (0.8)	4.6 (0.5)	3.7 (1.3)
	*Mean*	4.44 (0.40)	4.58 (0.39)	4.33 (0.50)
VAS	*Mean*	8.00 (1.75)	8.76 (2.00)	6.64 (1.41)

The eQUEST 2.0 scores range from 1 (not satisfied at all) to 5 (very satisfied), and the VAS scores range from 0 (not at all satisfied) to 10 (absolutely satisfied). The standard deviation (SD) is given in parenthesis. Results of the most important features per system are printed in bold.

In the eQUEST 2.0, only “speed” received scores below 4 (quite satisfied) for all three systems. The items that were rated as most important by the study participants were “effectiveness” (*n* = 6), “reliability” (*n* = 3), and “speed” (*n* = 3) for the hydrogel-based system; “speed” (*n* = 6), ”effectiveness” (*n* = 4), and ”learnability” (*n* = 4) for the tap water-based system; “speed” (*n* = 6), “effectiveness” (*n* = 5), and “easy of use,” “reliability” and “learnability” (all three: *n* = 3) for the dry electrode-based system.

Participants commented negatively on the unaesthetic and tight design of the caps and the low speed. On the other hand, most of the participants were positively surprised that it worked at all.

## 4. Discussion

Building a reliable BCI is possible with all the introduced EEG amplifier systems. However, small but important differences between the systems are detectable and deliver arguments to define special areas of application for each system.

### 4.1. Technical evaluation

For EEG measurements, it is crucial to have minimal noise resulting in a maximum signal-to-noise ratio. Having in mind that all short circuit RMS noise levels stayed below 1 μV, our measurements indicate that the short circuit RMS noise level of the tap water-based system is almost half the level of the hydrogel-based and less than half of the dry electrode-based system. It is obvious that the histogram of the tap water-based system is very narrow compared to the others, which means that the noise amplitude is low (see Figure 4). This is not surprising with the knowledge that the other electrodes are active electrodes, i.e., powered electronics are contained within the electrode, and this feature is probably the source of the additional short circuit noise. The active electronic parts (dry electrode-based and gel-based systems) and the active shielding technique (tap water-based system) are used to reduce noise pickup from cables. From our results, we cannot determine which technique works better regarding suppressing cable movement artifacts, because all the cables were fixed and not moving like they could in real-world usage. To determine the real-world behavior of the systems, we performed the user-centered evaluation.

### 4.2. User-centered evaluation

#### 4.2.1. Effectiveness and efficiency

Both “wet” systems, the hydrogel-based and the tap water-based, showed comparable averaged accuracies and seemed to be equally effective (see Figure 5). In addition, the increase of accuracy with increased number of sequences is also comparable (see Figure 6). However, the tap water-based as well as the dry electrode-based system showed a higher standard deviation of the accuracies (see Table 2). One possible explanation for this is that the connection between the electrode and the skin of the head is also a crucial factor. The shape of the human head is neither a sphere nor identical for all people. Therefore, the connection between the electrode and the head has to be very flexible. The tap water-based system as well as the dry electrode-based system has a more or less rigid connection. The dry electrode-based system with its gold alloy pins is delivered with two different pin lengths and three different cap sizes to be adaptable to different head shapes and hair lengths. It is time consuming to find a tradeoff between too much pressure of the pins against the skin (good signal quality, but less wearing comfort) and too little pressure (moderate signal quality, but comfortable). Since the time of our participants was limited and every participant used the system only once, we might have not found the optimal pin length and cap size solution for all of our participants. However, a visual inspection of the recorded signal before the measurement guaranteed that at least the alpha wave (i.e., an oscillation of approx. 8–13 Hz) was visible within the EEG, when the participants were instructed to close their eyes and relax.

The cotton pieces that connect the electrode of the tap water-based system with the skin are soft and flexible. However, they are rolled up and put into a housing where just 3 mm of the material is outside. Only these 3 mm of the material are available to fit the electrode to the head shape (see Figure 1). Consequently, the hair under each electrode has to be carefully pushed to the side (i.e., under the electrode housing of the cap) to reach a high real-world signal quality.

These problems will not occur with hydrogel-based electrodes, because the electrodes and the skin are connected with gel. Gel perfectly bridges the gap between the electrode and the skin. In addition, this connection is flexible, which means that the connection will not be lost if the head is slightly moved.

The described electrode fitting problem might also be an explanation for the higher inter-individual variances (i.e., higher standard deviations) of the tap water-based and the dry electrode-based systems (see Table 2).

Another shortcoming of the tap water-based system is that all 32 available electrodes are permanently connected to the amplifier in contrast to the two other systems where only the used number of electrodes are connected. However, it is possible to order the tap water-based system with fewer permanently connected electrodes. Nevertheless, the problem is that unused electrodes could swing around, when the user moves the head, and the weight of the cable bundle might pull the used electrodes down causing EEG artifacts. Therefore, we fastened the cable bundle and the unused electrodes to the cap to minimize those ar tifacts.

#### 4.2.2. Satisfaction

The high average accuracies achieved with the hydrogel-based and the tap water-based systems are also reflected in the results of the VAS and eQUEST 2.0. A mean VAS score of 8.00 (hydrogel-based) and 8.76 (tap water-based) and a mean eQUEST 2.0 score close to the maximum indicate that the participants were “very satisfied” with these two systems.

Although the mean eQUEST 2.0 score of the dry electrode-based system is not considerably lower than the scores of the two other systems, the negative difference of the VAS score is 2.12 to the tap water-based and 1.36 to the hydrogel-based systems (see Table 4). The main reason for that is probably the dissatisfaction of the users with the speed and effectiveness of the dry electrode-based system. Both criteria are rated low (below 4.0), whereas at the same time, they are listed as the most important features by most of the users.

However, the participants tested every system only once. Therefore, one can assume that the questionnaire scores may change when they are using the systems more often. Consequently, the results can only indicate a trend not absolute values.

One statement of the participants is consistent for all EEG amplifier systems: The users disliked the electrode cap. They felt that the cap was unaesthetic and conspicuous.

### 4.3. Comparison to existing literature

Volosyak et al. (2010) compared a prototype of the used water-based electrode system with passive Ag/AgCl electrodes. The major statements and conclusions out of this paper are “EEG activity can be measured with the novel water-based electrodes” and no significant differences between the two sensor modalities concerning the BCI classification accuracy (SSVEP spelling task) could be found. Both the points are also supported by our findings.

In Zander et al. (2011), the prototype of a dry electrode-based system was compared to an active Ag/AgCl electrode system. The electrodes were tested in two scenarios: ERPs were investigated and occipital alpha was measured. In addition, BCI classification accuracies were evaluated. The outcomes were, that no significant differences in the amplitude and the temporal structure of ERPs and no significant classification accuracy differences between the dry electrode-based and gel-based systems were detectable. However, the dry electrode-based system has a slightly lower ERP classification accuracy. Our findings indicate that the dry electrode-based system has a considerably lower ERP classification accuracy.

Guger et al. (2012) tested dry electrodes that were identical with the electrodes tested in this manuscript. Participants performed a simple P300 spelling task. The results were compared with the results gathered from standard passive and active electrodes. In addition, the dry electrodes were evaluated concerning the wearing comfort. Our results support their findings that the dry electrodes have a lower ERP classification accuracy. However, our assessed classification accuracy of the dry electrodes is on average more than 15% (cf. Guger et al.: 0.6%) lower and some participants reported discomfort after some time of usage. This is hardly surprising, considering that the participants of Guger et al. just had to copy spell five characters. In contrast, our participants had to copy spell at least 40 characters.

## 5. Conclusion

On the basis of the findings, the gel- and tap water-based systems are comparably suitable to build a very effective and efficient BCI. However, many users do not want to have gelled or wet hair and may accept a possibly lower effectiveness or efficiency to avoid inconveniences. Therefore, the dry based-electrode system is perfectly suitable.

Taking into account the outcome of a recent user-centered BCI evaluation (Kübler et al., 2014) and the recommendations of the BNCI roadmap (Brunner et al., 2015), the further development of BCI-suitable EEG acquisition systems should focus on the integration of the hardware into a single unit, wireless data transmission, and especially an appealing solution for placing gel-free electrodes on the head. The realization of these recommendations would strongly increase the user acceptance of BCIs outside the laboratory.

## Author contributions

AP and SW conceived the experiments. AP and JF implemented the data acquisition system. AP collected the data and performed all data analysis. AP, SW, JF, and GM wrote the manuscript.

## Funding

The research leading to these results has received funding from the European Community's, Seventh Framework Programme FP7/2007-2013, BackHome project grant agreement number 288566.

### Conflict of interest statement

The authors declare that the research was conducted in the absence of any commercial or financial relationships that could be construed as a potential conflict of interest.
